# Drug resistance patterns and genotype associations of *Trichomonas gallinae* in meat pigeons (*Columba livia*): insights from Guangdong Province, China

**DOI:** 10.3389/fvets.2023.1343321

**Published:** 2024-01-09

**Authors:** Haiming Cai, Yu Liu, Yibin Zhu, Siyun Fang, Dingai Wang, Zhuanqiang Yan, Hanqin Shen, Shenquan Liao, Nanshan Qi, Juan Li, Xuhui Lin, Junjing Hu, Yongle Song, Xiangjie Chen, Lijun Yin, Jianfei Zhang, Minna Lv, Mingfei Sun

**Affiliations:** ^1^Key Laboratory of Livestock Disease Prevention of Guangdong Province, Key Laboratory of Avian Influenza and Other Major Poultry Diseases Prevention and Control, Ministry of Agriculture and Rural Affairs, Institute of Animal Health, Guangdong Academy of Agricultural Sciences, Guangzhou, China; ^2^Wen's Group Academy, Wen's Foodstuffs Group Co., Ltd., Xinxing, Guangdong, China; ^3^Guangdong Jingjie Inspection and Testing Co., Ltd., Xinxing, Guangdong, China

**Keywords:** avian trichomoniasis, *Trichomonas gallinae*, drug resistant, prevalence, Guangdong Province

## Abstract

Avian trichomoniasis, caused by the protozoan parasite *Trichomonas gallinae*, is a prevalent and economically significant disease in pigeons. This study investigated the drug resistance of *T. gallinae* isolates in Guangdong Province, China. The results revealed that 25.3% (20/79) of the isolates were resistant to one or more of the four nitroimidazole drugs tested, namely, metronidazole, dimetridazole, secnidazole, and tinidazole. Secnidazole elicited the highest resistance rate (19.0%; 15/79), followed by tinidazole (17.7%; 14/79), metronidazole (17.7%; 14/79), and dimetridazole (13.9%; 11/79). An enormous majority of the resistant isolates (70.0%; 14/20) exhibited resistance to multiple drugs. Additionally, the resistance rate was significantly higher in isolates from birds aged < 30 days (53.3%; 8/15) than in those from older birds (23.1%; 12/52). Moreover, no drug resistance was detected in female pigeons. The genotype of the isolated strain was also associated with drug resistance. Specifically, 50.0% (15/30) of ITS-B genotypes exhibited resistance to drugs, while only 10.2% (5/49) of ITS-A genotypes demonstrated resistance. This study also found the growth characteristics of different *Trichomonas* isolates to be influenced by their genotypes and initial inoculum concentrations. These findings underscore the urgent need for effective measures to control and prevent drug-resistant *T. gallinae* infections in pigeons, thus ensuring the stable development of the pigeon industry.

## 1 Introduction

The pigeon industry has experienced remarkable growth in recent years, emerging as a thriving and economically significant sector. Pigeon breeding, in particular, has gained recognition as a valuable means of generating cash income, especially for vulnerable populations, such as impoverished women and educated unemployed youth, during challenging times (1). The demand for pigeon meat, renowned for its delicacy and nutritional value (2), has surged, prompting widespread commercial breeding in economically developed and developing countries across the globe, such as Australia (3), Spain (4), Italy (5), China (6), India (7), Bangladesh (8), Sri Lanka (9), and Egypt (10). Notably, China stands as the largest global producer of squabs, with an annual output of approximately 680 million, accounting for 80% of the global production (11). Therefore, the stable development of the pigeon industry is of paramount importance to ensuring the quality of life of the nation's citizens and sustaining economic growth.

The expansion of the pigeon industry has encountered various challenges, and one significant threat is avian trichomoniasis caused by the protozoan parasite *Trichomonas gallinae*. Studies conducted in different regions have highlighted the prevalence of *T. gallinae* in pigeons. For instance, Arfin et al. (12) investigated pigeon breeding farms in Jessore District, Bangkok, and found a 60% *T. gallinae* positivity rate. Similarly, Saikia et al. (13) examined domestic pigeons in the Assam area of India, reporting a *T. gallinae* positivity rate of 26.85% and a remarkably high rate of 56.25% in squabs. Jiang et al. (14) conducted a survey in Shandong Province, revealing an infection rate of 33.8% in pigeons. Feng et al. (15) surveyed pigeon farms in Beijing and observed that 28.3% of the pigeons were infected with *T. gallinae*. In our previous study, we detected an overall 26.6% (169/636) prevalence of *T. gallinae* infection in southern China (16).

*T. gallinae* primarily parasitizes the upper digestive tract of pigeons, leading to symptoms such as apathy, depression, and diarrhea. In severe cases, the infection can cause the formation of granulomatous lesions in the front digestive tract, resulting in the obstruction of the esophageal lumen and potentially leading to bird mortality owing to severe hunger (17, 18). In some cases, hosts may be asymptomatically infected with *T. gallinae*, displaying no apparent clinical signs. The variation in clinical symptoms among the infected hosts can be attributed to differences in susceptibility across pigeon species. For instance, high prevalence of *T. gallinae* have been documented in domestic pigeon (*C. livia*), with <1% of infected individuals exhibiting clinical signs (19, 20). Conversely, trichomonosis was identified as a significant factor contributing to the mortality of the pink pigeon (*Nesoenas mayeri*) (21, 22). Furthermore, the virulence of *T. gallinae* varies among its genotypes. Two genotypes, ITS-OBT-Tg-1 and ITS-OBT-Tg-2, have been previously identified, with the former associated with higher pathogenicity and the latter with mild or no pathogenicity (23, 24). This variation in pathogenicity appears to correlate with both the host species and the genotypes of *T. gallinae*. However, it remains important not to overlook the necessity of treating animals that exhibit symptoms of infection. Therefore, the current risk of prevention and control of *T. gallinae* in poultry cannot be ignored.

Currently, no prophylactic vaccine for avian trichomoniasis is available, and the main treatment approach relies on nitroimidazole drugs, such as metronidazole (25), dimetridazole (25, 26), secnidazole (27), and tinidazole (28). Nitroimidazole drugs exert its cytotoxic effects by inducing DNA damage within target cells through the reduction of its nitro group by one electron, resulting in the formation of a highly reactive and toxic radical anion (29). Although there have been ongoing studies discovering the therapeutic potential of plant essential oils against *T. gallinae*, such as essential oil extracted from *Pelargonium roseum* (30), *Cymbopogon flexuosus* (30), or Lamiaceae plants (31), these treatments have not yet received approval for clinical use. Currently, different countries have different treatment requirements for *T. gallinae*. For example, while the use of metronidazole and dimetridazole in food-producing animals has been banned in the United States (32) and the European Union (33, 34), these drugs can still be used as treatment options for other non-food-producing animals such as racing pigeons (35). In China, dimetridazole and metronidazole are permitted as therapeutic drugs in animals, but their substances should not be detectable in animal food (36). On the other hand, the use of ronidazole and tinidazole is prohibited in food-producing animals (37). The recommended dosages for these species are metronidazole at 60 mg/kg body weight and dimetridazole at 50 mg/kg body weight, administered orally or in drinking water at a concentration of 0.05% for 5–6 days (38). However, reports of drug resistance in trichomonads have raised concerns regarding the effectiveness of these drugs in treating avian trichomoniasis.

To address these concerns, this study aimed to investigate the prevalence of drug resistance in *T. gallinae* isolates in Guangdong Province, China. By examining the resistance rates of *T. gallinae* to nitroimidazole drugs, namely, metronidazole, dimetridazole, secnidazole, and tinidazole, we sought to provide insight into the extent and patterns of drug resistance in this region. Additionally, we explored the association between drug resistance and factors such as bird age and isolated-strain genotype. Understanding the prevalence and characteristics of drug-resistant *T. gallinae* strains is crucial for developing effective measures that control and prevent infections, thus safeguarding the stability and sustainable growth of the pigeon industry.

## 2 Materials and methods

### 2.1 Isolate collection and culture

In the present study, *T. gallinae* isolates were isolated from five cities in Guangdong Province, China, for drug-sensitivity testing. A total of 79 isolates were collected, and their origin and genotype information are listed in Supplementary Table S1. All strains were cultured in tryptone/yeast extract/maltose medium (TYM) supplemented with 10% fetal bovine serum (Gibico, USA), penicillin (Sigma-Aldrich, USA), streptomycin (Sigma-Aldrich, USA), and amphotericin B (Sigma-Aldrich, USA). The cultures were incubated in an anaerobic atmosphere using the sealed culture bag C-31 (Mitsubishi Gas Chemical Co., Inc, Japan) with AnaeroPack C-01 (Mitsubishi Gas Chemical Co., Inc, Japan) at 37°C. When in the logarithmic phase of growth, the proportion of active trophozoites was estimated by counting the number of active and non-active cells using standard light microscopy at 100× magnification. Strains with >95% mobility were used for further analysis.

### 2.2 Nitroimidazole-susceptibility profile

Under anaerobic conditions, the sensitivity of *T. gallinae* to nitroimidazoles was determined using the standard minimum lethal concentration (MLC) protocol, as described by Rouffaer et al. (39). In brief, 1 × 10^5^ trophozoites of different isolates were added to individual wells of a 96-well cell culture plate containing TYM medium supplemented with 10% fetal bovine serum and different concentrations (range: 0.2–400 μg/mL) of metronidazole, dimetridazole, secnidazole, or tinidazole, in triplicate. The control group received 0.1% dimethyl sulfoxide instead of the drugs. After incubation under anaerobic conditions at 37°C for 48 h, the number and activity of trophozoites were observed under a LEICA DMi8 inverted microscope at 400× magnification. The MLC was defined as the lowest concentration of metronidazole at which no motile trophozoites were observed. According to previous study (39), *T. gallinae* isolates with an anaerobic MLC ≥ 15.6 μg/mL are considered resistant to nitroimidazoles.

### 2.3 Cloning and sequencing of rDNA sequences

After collecting *T. gallinae* isolates via *in vitro* culture and thorough washings with phosphate-buffered saline, genomic DNA was extracted from all isolates using the QIAamp DNA Stool Mini Kit, according to the manufacturer's instructions. The concentration and purity of DNA were assessed with the Micro Drop Ultra Micro Spectrophotometer (Bio-DL, TX, USA). The extracted DNA was subsequently stored at a temperature of −40°C. Thereafter, the rDNA sequences (18S rRNA/ITS1-5.8S rRNA-ITS2) were amplified from the genomic DNA of each *T. gallinae* isolate using the polymerase chain reaction (PCR) with the primers, TgaTrend-F (5′-ACTGCCGGAGAAGGCGCCTGAG-3′) and TgaTrend-R (5′-TAAGACGTTGGGCTGTTCCCTT-3′), which described in our previous work (16). The primers were synthesized by Guangzhou IGE Biotechnology Co., Ltd. The reaction mixture for each PCR contained 100 ng of genomic DNA template, 0.3 μM of each primer, 25 μL of 2× KOD FX PCR buffer, 10 μL of 2 mM dNTPs, and 1 μL of KOD FX polymerase (Toyobo Co., Ltd., Japan), with double-distilled water added to achieve a final volume of 50 μL. The PCR program was performed with an initial denaturation at 95°C for 3 min, followed by 35 cycles of denaturation at 95°C for 30 s, annealing at 55°C for 30 s, and extension at 72°C for 1 min. A final extension step was performed at 72°C for 10 min. PCR products were analyzed using agarose gel electrophoresis, and the positive amplification products were purified and submitted to Guangzhou IGE Biotechnology Company for gene sequencing and sequence assembly.

### 2.4 Phylogenetic tree analyses

The rDNA sequencing results of the isolates were aligned with the reference sequences of the *T. gallinae* ITS1-5.8S rRNA-ITS2 and 18S rRNA regions using BioEdit 7.0 software. Phylogenetic analyses of the aligned sequences were conducted using Molecular Evolutionary Genetics Analysis (MEGA X) software (40). A neighbor-joining (NJ) tree was constructed with 1,000 bootstrap replications. The Maximum Composite Likelihood method (41) was employed to compute evolutionary distances, assuming homogeneous/uniform rates of transitions and transversions among lineages/sites. In pairwise sequence comparisons, positions containing alignment gaps and missing data were eliminated using the pairwise deletion option. Genotype identification was performed using references proposed by Grabensteiner et al. (42) and Gerhold et al. (43) along with several reference sequences for each genotype. The ITS (AY245137) and 18S (AF124609) lineages of *Pentatrichomonas hominis* were used as an outgroup for phylogenetic analysis. The nomenclature for ITS lineages of *T. gallinae* can be confusing due to multiple synonymous names. To provide clarity, we can establish a unified definition. ITS-A and ITS-B are equivalent to both ITS-IV and ITS-I, as described by Grabensteiner et al. (42), as well as ITS-A/B and ITS-D, as described by Gerhold et al. (43). Additionally, they correspond to ITS-OBT-Tg-1 and ITS-OBT-Tg-2, according to Martínez-Herrero et al. (41). And 18S-IV and 18S-VI adhere to the naming scheme proposed by Grabensteiner et al. (42).

### 2.5 Analysis of parasite growth curves

To investigate the growth-cycle changes of the *T. gallinae* isolates, their growth curves were monitored using the method described by Mohamed et al. (44). Prior to this, each isolate was sub-cultured in fresh TYM medium containing 10% fetal bovine serum for 24 h. The cell density of the parasites was subsequently determined using cell-counting chamber slides and further diluted to either 1 × 10^4^ or 1 × 10^5^ cells/mL. During growth-curve monitoring, time points were set at 12, 24, 48, 60, 72, 84, and 96 h. Thus, a total of 42 sample tubes were prepared for each isolate, each containing 1 mL of either 1 × 10^4^ or 1 × 10^5^ cells/mL parasite cells. All tubes were incubated anaerobically at 37°C until the corresponding time point, after which the samples were removed from the tubes for cell counting. To ensure accuracy, each time point was tested in triplicate.

### 2.6 Statistical analysis

The prevalence of *T. gallinae* infection in pigeons was analyzed using a generalized linear model with 95% confidence intervals. The relationship between *T. gallinae* infection in pigeons and risk factors such as age, sex, and biogeographical region was determined via statistical analysis using the χ^2^ test. This analysis was performed using SPSS 19.0 for Windows (SPSS Inc., USA).

## 3 Results

### 3.1 Analysis of *T. gallinae*-isolate resistance to nitroimidazole drugs in Guangdong Province

In this study, we analyzed drug resistance in 79 stable cultures of avian *Trichomonas* isolated from clinical cases in Guangdong province (Supplementary Table S1). The results revealed varying degrees of resistance among the isolates to four tested nitroimidazole drugs, with an overall resistance rate of 25.3% (20/79). Secnidazole elicited the highest resistance rate (19.0%; 15/79), while dimetridazole had the lowest (13.9%; 11/79). However, no significant difference in resistance rates was noted among the four drugs (χ^2^ = 0.804, df = 3, *P* = 0.848). Notably, only 30.0% (6/20) of the resistant isolates exhibited resistance to a single drug, while 70.0% (14/20) displayed multiple-drug resistance (Table 1).

**Table 1:** Drug resistance and multiple drug resistance profile of *Trichomonas gallinae* isolates from Guangdong Province to metronidazole, secnidazole, tinidazole, and dimetridazole.

**Drug category**	**Single drug Resistance^a^**	**Dual drug resistance^a^**	**Triple drug resistance^a^**	**Quadruple drug resistance^a^**	**Total drug resistance^b^**	**Total drug sensitive^b^**	**Total number of isolates**
Tinidazole	3	0	1	10	14	65	79
Secnidazole	2	1	2	10	15	64	
Metronidazole	1	1	2	10	14	65	
Dimetridazole	0	0	1	10	11	68	

a Drug resistance is classified into distinct levels, namely single drug resistance, dual drug resistance, triple drug resistance, and quadruple drug resistance. The numerical values in the data table indicate the number of isolates that exhibit resistance to a specific number of drugs within each corresponding drug category.

b Total drug resistance column indicates the total number of isolates exhibiting drug resistance (single, dual, triple, or quadruple) for each corresponding drug listed in the row. Total drug sensitive column reports the total number of isolates that are free from any level of drug resistance for the corresponding drugs listed in the row.

### 3.2 Analysis of the association of host region, age, and sex with drug resistance phenotype

We conducted a statistical analysis of the drug resistance rates of *T. gallinae* isolates to nitroimidazole drugs in different regions, and the specific results are shown in Table 2. These rates varied across different regions: in Zhaoqing, Jiangmen, Qingyuan, Chaozhou, and Zhanjiang, the rates were 33.3% (1/3), 0.0% (0/19), 0.0% (0/1), 53.8% (7/13), and 27.9% (12/43), respectively. In these same regions, the rates of multiple-drug resistance were 0.0% (0/1), 0.0% (0/0), 0.0% (0/0), 71.4% (5/7), and 75.0% (9/12), respectively. Data for isolates with zero resistance were excluded from the statistical analysis, and the results showed significant differences in resistance rates among different regions (χ^2^ = 12.631, df = 4, *P* = 0.013). However, no significant difference in multiple-drug resistance rates was observed among the resistant isolates (χ^2^ = 2.483, df = 2, *P* = 0.289).

**Table 2:** Drug resistance and genotypic analysis of *Trichomonas gallinae* isolates from Guangdong Province to metronidazole, secnidazole, tinidazole, and dimetridazole.

**Drug category^a^**	**ITS-A/18S-VI**		**ITS-B/18S-IV**	
	**Resistance**	**sensitive**	**Resistance**	**sensitive**
Tinidazole	2	47	13	17
Secnidazole	3	46	13	17
Metronidazole	3	46	12	18
Dimetridazole	1	48	11	19
Total	5	44	15	15

a The “Total” count in the row includes all isolates that exhibit resistance or sensitivity to any of the four drugs (Tinidazole, Secnidazole, Metronidazole, and Dimetridazole) among the ITS-A/18S-VI or ITS-B/18S-IV genotypes.

The drug resistance rates of *T. gallinae* isolates were significantly influenced by host age, as shown in Table 2. The resistance rates by age were as follows: 53.3% (8/15), 0.0% (0/12), and 23.1% (12/52) for birds aged < 30, 31–180, and >180 days, respectively. The multiple-drug resistance rates were 75.0% (6/8), 0.0% (0/0), and 66.7% (8/12) for birds aged < 30, 30–180, and >180 days, respectively. Data for isolates with zero resistance were excluded from the statistical analysis, thus revealing considerably significant differences in drug resistance rates across various host ages (χ^2^ = 10.433, df = 2, *P* = 0.005). Although multiple-drug resistance rates were high in all age groups, the difference was not statistically significant (χ^2^ = 0.159, df = 1, *P* = 0.690).

Owing to a lack of sex-related statistics during sampling, only nine *T. gallinae* isolates had their host sex determined in this experiment. The single-drug and multiple-drug resistance rates for these nine isolates are shown in Table 3: for female pigeons, the single-drug and multiple-drug resistance rates were 0.0% (0/6) and 0.0% (0/0), respectively; for male pigeons, the single-drug and the multiple-drug resistance rates were 33.3% (1/3) and 0.0% (0/1), respectively. As the *T. gallinae* isolates from female pigeons did not exhibit laboratory drug resistance, statistical analysis could not be performed on them.

**Table 3:** Nitroimidazole resistance profile of *Trichomonas gallinae* isolates from Guangdong Province.

**Independent variable**	**Variable**	**No. of isolate**	**Drug resistance level** ^**a**^					**Drug-resistant isolate (*n*)**	**Resistance rate (%)^b^**	**95 % *CI***	**Multidrug-resistant isolate (*n*)**	**Multidrug resistance rate (%)^c^**	**95 % *CI***
			**0**	**1**	**2**	**3**	**4**						
Location	Zhaoqing	3	1	1	0	0	0	1	33.3	1.76–87.47	0	0.0	0.00–94.54
	Jiangmen	19	19	0	0	0	0	0	0.0	0.00–20.92	0	0.0	/
	Qingyuan	1	0	0	0	0	0	0	0.0	0.00–94.54	0	0.0	/
	Chaozhou	13	6	2	0	2	3	7	53.8	26.12–79.60	5	71.4	30.26–94.89
	Zhanjiang	43	31	3	1	0	8	12	27.9	15.84–43.91	9	75.0	42.84–93.31
Age	Squab (< 30 d)	15	7	2	0	2	4	8	53.3	27.42–77.72	6	75.0	35.58–95.55
	Young pigeon (30–180 d)	12	12	0	0	0	0	0	0.0	0.00–30.13	0	0.0	/
	Adult pigeon (>180 d)	52	40	4	1	0	7	12	23.1	12.98–37.18	8	66.7	35.44–88.73
Sex	Female	6	6	0	0	0	0	0	0.0	0.00–48.32	0	0.0	/
	Male	3	2	1	0	0	0	1	33.3	1.76–87.47	0	0.0	0.00–94.54
Total		79	59	6	1	2	11	20	25.3	16.50–36.57	14	70.0	45.67–87.16

a 0, sensitive strain; 1, single resistant strain; 2, double resistant strain; 3, triple resistant strain; 4, quadruple resistant strain.

b Resistance rate (%) = (number of resistant strains/total number) × 100.

c Multidrug resistance rate (%) = (number of multidrug-resistant strains/number of resistant strains) × 100.

### 3.3 Association analysis between genotype and drug resistance phenotype

**Figure 1:**
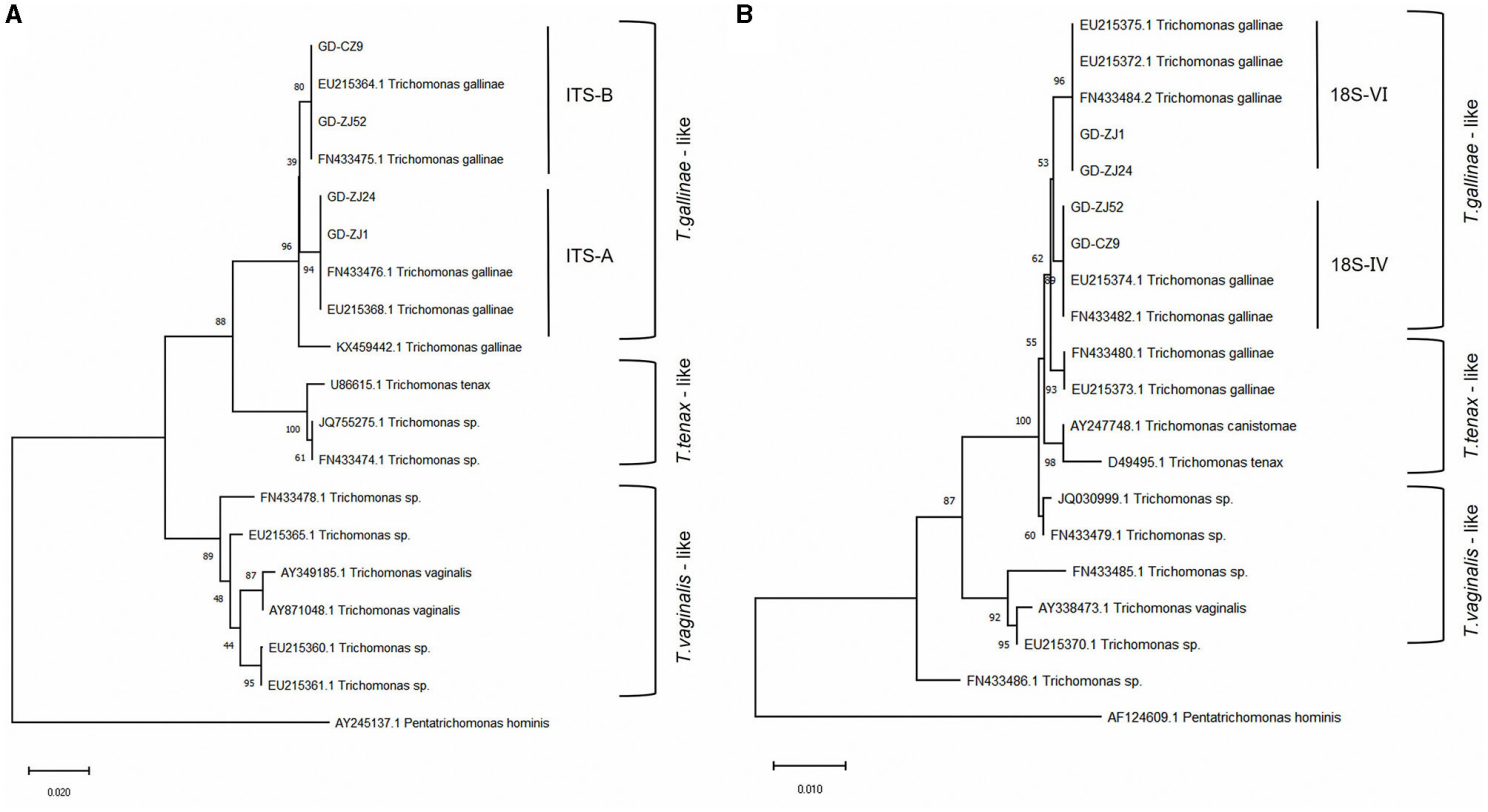
Genetic evolutionary analysis of four typical *Trichomonas gallinae* isolates based on the ITS1/5.8S/ITS2 genes **(A)** and 18S rRNA genes **(B)**. Phylogenetic analysis was performed using MEGA-X software with the neighbor-joining method and 1,000 replications as well as the best-fit model. The accession numbers of the reference sequences can be retrieved from GenBank.

We conducted sequencing of the ITS region for 79 clinical isolates of *T. gallinae*. Phylogenetic analysis revealed the presence of two genotypes in all isolates (Figure 1): ITS-A/18S-VI (also known as ITS-OBT-Tg-1) and ITS-B/18S-IV (also known as ITS-OBT-Tg-2). Among the isolates, 49 belonged to the ITS-A/18S-VI genotype, while 30 belonged to the ITS-B/18S-IV genotype. On analyzing the correlation between drug resistance and genotype, we observed that 10.2% (5/49) of ITS-A genotypes were drug-resistant strains, whereas 50.0% (16/30) of ITS-B genotypes exhibited drug resistance. A significant difference in drug resistance was noted between the two *T. gallinae* genotypes (χ^2^ = 15.586, df = 1, *P* = 0.000). Both A and B genotypes exhibited the greatest resistance to secnidazole, with resistance rates of 6.1% (3/49) and 43.3% (13/30), respectively. However, no significant differences were observed between the two genotypes in terms of resistance to the four individual tested drugs (A: χ^2^ = 1.281, df = 3, *P* = 0.734; B: χ^2^ = 0.379, df = 3, *P* = 0.944) (Table 3).

### 3.4 Growth curves of four clinically typical isolates in the TYM culture medium

**Figure 2:**
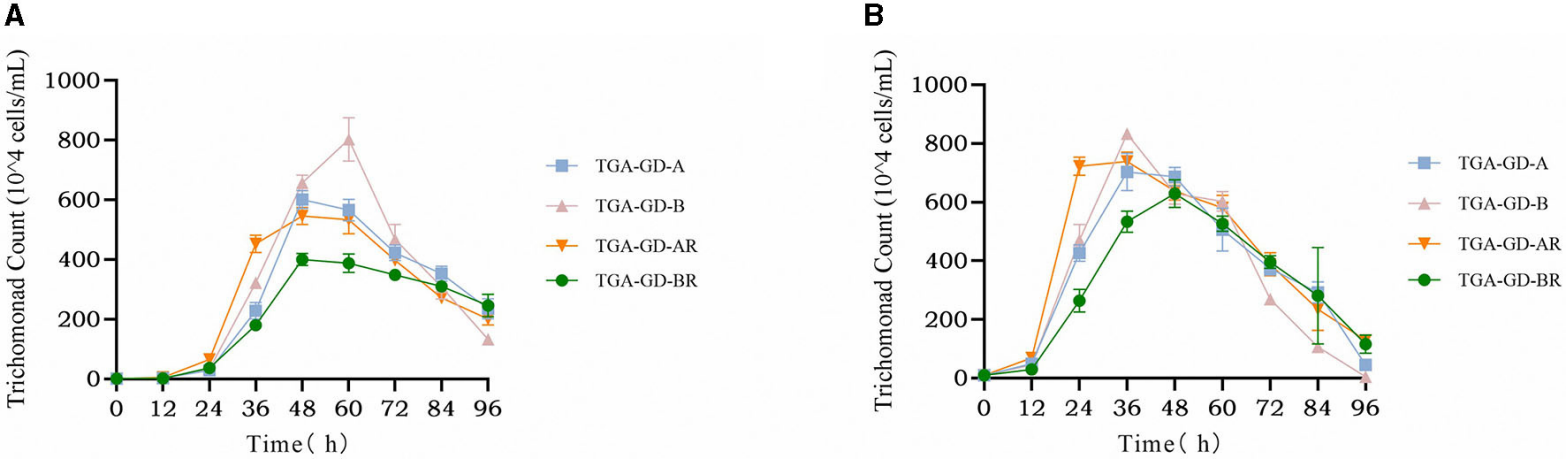
Growth curves of different *Trichomonas gallinae* isolates at two different initial inoculum concentrations: 1 × 10^4^ cells/mL **(A)** and 1 × 10^5^ cells/mL **(B)**. In both figures, the *x*-axis represents the time in hours, and the *y*-axis represents the cell density in cells/mL. Each curve corresponds to a different *T. gallinae* isolate: TGA-GD-A (ITS-A genotype sensitive strain), TGA-GD-AR (ITS-A genotype drug-resistant strain), TGA-GD-B (ITS-B genotype sensitive strain), and TGA-GD-BR (ITS-B genotype drug-resistant strain).

We analyzed the growth curves of four clinically typical isolates: TGA-GD-A (ITS-A genotype sensitive strain), TGA-GD-AR (ITS-A genotype drug-resistant strain), TGA-GD-B (ITS-B genotype sensitive strain), and TGA-GD-BR (ITS-B genotype drug-resistant strain). The growth curves were determined in the TYM culture medium at various initial inoculum concentrations, and the results are presented in Figure 2. At an initial inoculum concentration of 1 × 10^4^ cells/mL, TGA-GD-A, TGA-GD-BR, and TGA-GD-AR exhibited growth lags before entering the logarithmic phase, which occurred between 12 and 48 h. During this period, the trichomonads grew rapidly and reached their peak at 48 h. After 48 h, all trichomonads entered the death phase, characterized by a decline in cell viability and an increase in the number of dead cells. TGA-GD-B reached its growth peak at 60 h, with a logarithmic growth period between 12 and 60 h, followed by the death phase after 60 h. At their growth peaks, these strains differed in their maximum cell densities, which were as follows: 6.01 × 10^6^, 5.46 × 10^6^, 8.02 × 10^6^, and 4.00 × 10^6^ cells/mL for TGA-GD-A, TGA-GD-AR, TGA-GD-B, and TGA-GD-BR, respectively. At an initial inoculum concentration of 1 × 10^5^ cells/mL, all isolates exhibited a growth lag before entering the logarithmic phase between 12 and 36 h. Subsequently, they entered the death phase after 36 h, except for TGA-GD-BR, which reached its growth peak at 48 h and gradually declined thereafter. At their growth peaks, the strains' maximum cell densities were as follows: 7.03 × 10^6^, 5.33 × 10^6^, 8.33 × 10^6^, and 6.38 × 10^6^ cells/mL for TGA-GD-A, TGA-GD-AR, TGA-GD-B, and TGA-GD-BR, respectively. These results indicate that the growth characteristics of different *Trichomonas* isolates are influenced by their genotypes and initial inoculum concentrations.

## 4 Discussion

*T. gallinae* is a flagellated protozoan that primarily infects the upper digestive tract of birds, leading to inflammatory reactions and potential mortality. Nitroimidazole drugs have been widely used as the first-line treatment for trichomoniasis in birds. However, increasing reports indicate that *T. gallinae* has developed resistance to these drugs, posing a significant challenge to their effectiveness. Franssen and Lumeij (45) reported treatment failures in racing pigeons and conducted the first *in vitro* drug sensitivity test, confirming the emergence of resistance. Subsequent investigations conducted by Munoz et al. (25) revealed varying efficacies among different nitroimidazole drugs in trichomoniasis treatment, with metronidazole exhibiting the greatest resistance factors. In a separate study, Zimre-Grabensteiner et al. (46) found higher sensitivity to nitroimidazole drugs in *T. gallinae* isolated from wild birds compared to those from domesticated pigeons, possibly due to sublethal selection pressure during domestication. This study on drug resistance in *T. gallinae* isolates in Guangdong Province provides valuable insights into the prevalence, patterns, and factors influencing drug resistance in avian trichomoniasis.

The current study investigated nitroimidazole drug sensitivity screening of 79 clinical *T. gallinae* isolates obtained from an epidemiological survey. The results revealed varying degrees of resistance to different nitroimidazole drugs, with secnidazole exhibiting the lowest sensitivity and dimetridazole and tinidazole eliciting relatively higher sensitivities. Notably, resistance to nitroimidazole drugs went beyond a single drug, involving varying degrees of resistance to multiple drugs. The increasing rate of multiple-drug resistance is concerning, suggesting that *T. gallinae* is becoming progressively challenging to treat with these drugs. Considering the rising resistance, alternative therapeutic options may be necessary. Recent *in vitro* studies have demonstrated promising potential in various natural compounds against trichomonads, including medicinal plant extracts (47, 48), possibly opening novel avenues for drug development. Further clinical and pharmacokinetic studies that evaluate their potential practical use are warranted.

This study also revealed significant differences in resistance rates across various regions within Guangdong Province. The resistance rates ranged from 0.0 to 53.8% across different regions, with multiple-drug resistance rates also varying. Over 30% of isolates from certain areas, such as Zhaoqing and Chaozhou, demonstrated drug resistance, underscoring the imperativeness of investigating the factors contributing to resistance in these locations. As indicated by Bondad-Reantaso et al. (49), regional differences in drug resistance rates can be influenced by local antimicrobial usage practices, biosecurity measures, and strain variations. Further research is essential to verify whether similar mechanisms are applicable to *T. gallinae* infections in the cities mentioned above.

Host age was found to significantly influence drug resistance, with rates of 0.0, 53.3, and 23.1% observed in birds aged 31–180, < 30, and >180 days, respectively. This finding raises questions regarding the factors contributing to higher drug resistance in younger birds. In a separate study conducted by Akanbi et al. (50), 291 tissue samples from 237 necropsied carcasses submitted from 99 poultry flocks in northern-central Nigeria were analyzed to investigate *Escherichia coli* infections. The study revealed that 56% of *E. coli* isolates exhibited multidrug antibiotic resistance, with a higher prevalence observed in younger birds. These findings accentuate the need for further research that elucidates the mechanisms underlying drug resistance across different bird age groups.

Owing to limited sex-related statistics during sampling, a thorough analysis of the association between host sex and drug resistance was not possible. However, the intriguing observation that *T. gallinae* isolates from female pigeons did not demonstrate laboratory drug resistance deserves additional investigation with a larger sample size. Overall, these findings provide valuable insights into the prevalence of and factors associated with drug resistance in *T. gallinae* isolates in Guangdong Province. Further research is required to understand the mechanisms underlying resistance, explore the factors contributing to regional variations and age-related differences, and investigate the potential association between sex and drug resistance.

Additionally, different *T. gallinae* genotypes exhibited varying levels of resistance, with the ITS-B genotype displaying higher resistance than the ITS-A genotype. This observation is consistent with previous research findings suggesting that genetic differences across protozoan genotypes potentially impact drug susceptibility and treatment efficacy (51). This variation may also be related to the clinical treatment strategies employed. Martínez-Herrero et al. (52) found the ITS-A genotype with MLS types to have higher virulence and contribute to an increased risk of lesion development. The weaker pathogenicity of the ITS-B genotype may cause farmers to administer lower doses of medication more frequently, resulting in a higher prevalence of ITS-B drug-resistant strains in clinical settings. Notwithstanding, acknowledging the study's limitations, which include limited data and the absence of strain verification in animal models, preventing the drawing of definitive conclusions, is important. Nonetheless, the observed association between genotype and drug resistance highlights the potential for genotype-based therapies.

Growth-curve analysis of four *T. gallinae* isolates (TGA-GD-A, TGA-GD-AR, TGA-GD-B, and TGA-GD-BR) revealed distinct patterns influenced by genotype and initial inoculum concentration. At lower inoculum levels, TGA-GD-B had a markedly longer logarithmic growth phase and achieved higher maximum cell densities than the other ITS-A isolates. This aligns with previous findings by Martínez-Herrero et al. (24) who reported higher growth rates and cell yields for ITS-B isolates cultured in TYM medium. Intriguingly, while TGA-GD-AR exhibited reduced growth compared with the sensitive TGA-GD-A strain at both inoculum levels, the resistant ITS-B isolate TGA-GD-BR reached cell densities comparable to those of its sensitive counterpart TGA-GD-B. This indicates that the development of 5-nitroimidazole resistance impairs growth in ITS-A but not in ITS-B isolates. Further mechanistic studies are warranted to elucidate the molecular determinants of differential fitness impacts between genotypes. Varying the inoculum concentration modulated growth kinetics, with higher initial cell densities shortening the logarithmic phase duration. This self-limiting effect of inoculum size has widely been reported for *Trichomonas* and may emanate from rapid nutrient depletion in denser cultures (53). Overall, this analysis reveals key interactions among genotype, drug-resistance status, and inoculum concentration in shaping *T. gallinae* growth dynamics. The methodology and datasets provide a useful framework for dissecting the complex interplay between genetic and environmental factors governing trichomonad virulence and pathogenesis.

## 5 Conclusions

In summary, this study on drug resistance in *T. gallinae* isolates in Guangdong Province provides important insights into the prevalence, patterns, and factors influencing drug resistance in avian *Trichomonas* infections. The findings enhance our understanding of the underlying resistance mechanisms and emphasize the need for appropriate strategies to address this issue. Further research is required to explore the genetic mechanisms, regional variations, age-related differences, and growth characteristics associated with drug resistance in *T. gallinae*.

## Data availability statement

The original contributions presented in the study are publicly available. This data can be found here: https://www.ncbi.nlm.nih.gov/nuccore/; OR478490-OR478491.

## Ethics statement

The animal study was approved by the Animal Care and Use Committee of the Institute of Animal Health, Guangdong Academy of Agricultural Sciences. The study was conducted in accordance with the local legislation and institutional requirements.

## Author contributions

HC: Conceptualization, Formal analysis, Investigation, Writing – original draft. YL: Conceptualization, Formal analysis, Investigation, Writing – original draft. YZ: Conceptualization, Investigation, Methodology, Writing – original draft. SF: Conceptualization, Investigation, Methodology, Writing – original draft. DW: Conceptualization, Investigation, Methodology, Writing – original draft. ZY: Conceptualization, Investigation, Methodology, Writing – original draft. HS: Conceptualization, Investigation, Methodology, Writing – original draft. SL: Conceptualization, Investigation, Methodology, Writing – original draft. NQ: Conceptualization, Investigation, Methodology, Writing – review & editing. JL: Conceptualization, Investigation, Methodology, Writing – review & editing. XL: Methodology, Resources, Supervision, Writing – original draft. JH: Methodology, Resources, Supervision, Writing – original draft. YS: Methodology, Resources, Supervision, Writing – original draft. XC: Methodology, Resources, Supervision, Writing – original draft. LY: Methodology, Resources, Supervision, Writing – original draft. JZ: Methodology, Resources, Supervision, Writing – original draft. ML: Funding acquisition, Methodology, Project administration, Supervision, Writing – review & editing. MS: Funding acquisition, Methodology, Project administration, Supervision, Writing – review & editing.
