# Supplementary material for: Drug resistance patterns and genotype associations of Trichomonas gallinae in meat pigeons (Columba livia): insights from Guangdong Province, China
Source: Front Vet Sci. 2024 Jan 9;10:1343321. doi: 10.3389/fvets.2023.1343321 (PMC10803545; doi:10.3389/fvets.2023.1343321)
Supplement: Supplementary file 1 [file Data_Sheet_1.DOCX]

**Supplementary file:**

**Drug Resistance Patterns and Genotype Associations of *Trichomonas gallinae* in** **Meat Pigeons (*Columba livia*): Insights from Guangdong Province, China**

Haiming Cai ^1†^, Yu Liu ^1†^, Yibin Zhu ^1^, Siyun Fang ^2^, Dingai Wang ^2^, Zhuanqiang Yan ^2^, Hanqin Shen ^3^, Shenquan Liao ^1^, Nanshan Qi ^1^, Juan Li ^1^, Xuhui Lin ^1^, Junjing Hu ^1^, Yongle Song ^1^, Xiangjie Chen ^1^, Lijun Yin ^1^, Jianfei Zhang ^1^, Minna Lv ^1*^, Mingfei Sun ^1*^

1 Key Laboratory of Livestock Disease Prevention of Guangdong Province, Key Laboratory of Avian Influenza and Other Major Poultry Diseases Prevention and Control, Ministry of Agriculture and Rural Affairs, Institute of Animal Health, Guangdong Academy of Agricultural Sciences, Guangzhou, 510640, China; caihaiming@gdaas.cn (H.C.); LY2023@wchscu.cn (Y.L.)

2 Wen’s Group Academy, Wen’s Foodstuffs Group Co., Ltd., Xinxing, Guangdong 527400, China.

3 Guangdong Jingjie Inspection and Testing Co., Ltd., Xinxing, Guangdong 527400, China.

***** Correspondence: lvminna@gdaas.cn (M.L.); sunmingfei@gdaas.cn (M.S.)

† These authors contributed equally to this work.

Table S1. Nitroimidazole resistance profile of *Trichomonas gallinae* isolate in Guangdong Province

| Isolate ID | Age | City | Sex Type | Genetype | | MLC（μg/mL） | | | |
| --- | --- | --- | --- | --- | --- | --- | --- | --- | --- |
|  |  |  |  | Lineage by ITS | Lineage by 18S rDNA | TNZ | SCZ | MTZ | DMZ |
| GD-ZQ8 | 6d | ZhaoQing | ♀ | ITS-B | 18S-IV | 0 | 0 | 0 | 0 |
| GD-QY7 | 18d | Qingyuan | ♀ | ITS-B | 18S-IV | 12.5 | 12.5 | 12.5 | 12.5 |
| GD-ZQ10 | 25d | ZhaoQing | ♂ | ITS-B | 18S-IV | 25 | 12.5 | 12.5 | 12.5 |
| GD-ZQ11 | 25d | ZhaoQing | ♂ | ITS-B | 18S-IV | 12.5 | 12.5 | 12.5 | 12.5 |
| GD-CZ1 | 1y | Chaozhou | \ | ITS-B | 18S-IV | 50 | 50 | 25 | 25 |
| GD-CZ9 | 4y | Chaozhou | \ | ITS-B | 18S-IV | 0 | 0 | 0 | 0 |
| GD-CZ11 | 30d | Chaozhou | \ | ITS-B | 18S-IV | 12.5 | 12.5 | 12.5 | 12.5 |
| GD-CZ12 | 30d | Chaozhou | \ | ITS-B | 18S-IV | 12.5 | 25 | 25 | 25 |
| GD-CZ13 | 30d | Chaozhou | \ | ITS-B | 18S-IV | 25 | 25 | 25 | 12.5 |
| GD-CZ15 | 3y | Chaozhou | \ | ITS-B | 18S-IV | 0 | 0 | 0 | 0 |
| GD-CZ16 | 3y | Chaozhou | \ | ITS-B | 18S-IV | 0 | 0 | 0 | 0 |
| GD-CZ17 | 3y | Chaozhou | \ | ITS-B | 18S-IV | 25 | 50 | 25 | 25 |
| GD-CZ18 | 3y | Chaozhou | \ | ITS-B | 18S-IV | 12.5 | 25 | 12.5 | 12.5 |
| GD-CZ19 | 3y | Chaozhou | \ | ITS-B | 18S-IV | 50 | 50 | 50 | 50 |
| GD-CZ20 | 3y | Chaozhou | \ | ITS-B | 18S-IV | 12.5 | 12.5 | 12.5 | 12.5 |
| GD-ZJ3 | 2y | Zhanjiang | \ | ITS-B | 18S-IV | 12.5 | 12.5 | 0 | 0 |
| GD-ZJ12 | 2y | Zhanjiang | \ | ITS-B | 18S-IV | 12.5 | 12.5 | 12.5 | 12.5 |
| GD-ZJ15 | 2y | Zhanjiang | \ | ITS-B | 18S-IV | 12.5 | 0 | 0 | 0 |
| GD-ZJ16 | 2y | Zhanjiang | \ | ITS-B | 18S-IV | 12.5 | 12.5 | 12.5 | 0 |
| GD-ZJ17 | 2y | Zhanjiang | \ | ITS-B | 18S-IV | 25 | 12.5 | 12.5 | 12.5 |
| GD-ZJ34 | 2y | Zhanjiang | \ | ITS-B | 18S-IV | 50 | 50 | 25 | 25 |
| GD-ZJ36 | 2y | Zhanjiang | \ | ITS-B | 18S-IV | 0 | 0 | 0 | 0 |
| GD-ZJ37 | 2y | Zhanjiang | \ | ITS-B | 18S-IV | 25 | 50 | 25 | 25 |
| GD-ZJ39 | 2y | Zhanjiang | \ | ITS-B | 18S-IV | 25 | 25 | 25 | 25 |
| GD-ZJ50 | 25d | Zhanjiang | \ | ITS-B | 18S-IV | 25 | 25 | 25 | 25 |
| GD-ZJ51 | 25d | Zhanjiang | \ | ITS-B | 18S-IV | 50 | 25 | 25 | 25 |
| GD-ZJ52 | 25d | Zhanjiang | \ | ITS-B | 18S-IV | 25 | 25 | 25 | 25 |
| GD-ZJ53 | 25d | Zhanjiang | \ | ITS-B | 18S-IV | 25 | 25 | 25 | 25 |
| GD-ZJ55 | 25d | Zhanjiang | \ | ITS-B | 18S-IV | 12.5 | 0 | 0 | 0 |
| GD-ZJ57 | 25d | Zhanjiang | \ | ITS-B | 18S-IV | 12.5 | 12.5 | 12.5 | 12.5 |
| GD-JM1 | 171d | Jiangmen | \ | ITS-A | 18S-VI | 12.5 | 12.5 | 0 | 0 |
| GD-JM3 | 1.43y | Jiangmen | \ | ITS-A | 18S-VI | 12.5 | 0 | 0 | 0 |
| GD-JM7 | 5.92y | Jiangmen | \ | ITS-A | 18S-VI | 0 | 0 | 0 | 0 |
| GD-JM17 | 33d | Jiangmen | \ | ITS-A | 18S-VI | 0 | 0 | 0 | 0 |
| GD-JM18 | 33d | Jiangmen | \ | ITS-A | 18S-VI | 12.5 | 0 | 0 | 0 |
| GD-JM22 | 33d | Jiangmen | \ | ITS-A | 18S-VI | 0 | 12.5 | 0 | 12.5 |
| GD-JM24 | 33d | Jiangmen | \ | ITS-A | 18S-VI | 12.5 | 0 | 0 | 0 |
| GD-JM25 | 33d | Jiangmen | \ | ITS-A | 18S-VI | 0 | 12.5 | 0 | 12.5 |
| GD-JM26 | 33d | Jiangmen | \ | ITS-A | 18S-VI | 0 | 0 | 0 | 0 |
| GD-JM27 | 33d | Jiangmen | \ | ITS-A | 18S-VI | 12.5 | 12.5 | 0 | 0 |

Table S1 (continued)

| Isolate ID | Age | City | Sex Type | Genetype | | MLC（μg/mL） | | | |
| --- | --- | --- | --- | --- | --- | --- | --- | --- | --- |
|  |  |  |  | Lineage by ITS | Lineage by 18S rDNA | TNZ | SCZ | MTZ | DMZ |
| GD-JM31 | 33d | Jiangmen | \ | ITS-A | 18S-VI | 0 | 12.5 | 0 | 12.5 |
| GD-JM33 | 33d | Jiangmen | \ | ITS-A | 18S-VI | 12.5 | 12.5 | 12.5 | 12.5 |
| GD-JM38 | 33d | Jiangmen | \ | ITS-A | 18S-VI | 0 | 0 | 0 | 0 |
| GD-JM39 | 33d | Jiangmen | \ | ITS-A | 18S-VI | 0 | 0 | 0 | 0 |
| GD-JM42 | 1.5y | Jiangmen | ♂ | ITS-A | 18S-VI | 12.5 | 12.5 | 0 | 12.5 |
| GD-JM43 | 1.5y | Jiangmen | ♀ | ITS-A | 18S-VI | 0 | 0 | 0 | 12.5 |
| GD-JM47 | 1.5y | Jiangmen | ♀ | ITS-A | 18S-VI | 12.5 | 12.5 | 0 | 12.5 |
| GD-JM50 | 1.5y | Jiangmen | ♀ | ITS-A | 18S-VI | 0 | 12.5 | 0 | 12.5 |
| GD-JM53 | 3y | Jiangmen | ♀ | ITS-A | 18S-VI | 12.5 | 12.5 | 12.5 | 12.5 |
| GD-CZ10 | 30d | Chaozhou | \ | ITS-A | 18S-VI | 12.5 | 25 | 12.5 | 12.5 |
| GD-CZ14 | 30d | Chaozhou | \ | ITS-A | 18S-VI | 0 | 0 | 0 | 0 |
| GD-ZJ1 | 2y | Zhanjiang | \ | ITS-A | 18S-VI | 0 | 0 | 0 | 0 |
| GD-ZJ2 | 2y | Zhanjiang | \ | ITS-A | 18S-VI | 12.5 | 0 | 12.5 | 0 |
| GD-ZJ7 | 2y | Zhanjiang | \ | ITS-A | 18S-VI | 25 | 12.5 | 12.5 | 12.5 |
| GD-ZJ8 | 2y | Zhanjiang | \ | ITS-A | 18S-VI | 0 | 0 | 0 | 0 |
| GD-ZJ9 | 2y | Zhanjiang | \ | ITS-A | 18S-VI | 12.5 | 12.5 | 0 | 0 |
| GD-ZJ10 | 2y | Zhanjiang | \ | ITS-A | 18S-VI | 12.5 | 25 | 25 | 0 |
| GD-ZJ11 | 2y | Zhanjiang | \ | ITS-A | 18S-VI | 12.5 | 0 | 0 | 0 |
| GD-ZJ13 | 2y | Zhanjiang | \ | ITS-A | 18S-VI | 12.5 | 12.5 | 12.5 | 12.5 |
| GD-ZJ14 | 2y | Zhanjiang | \ | ITS-A | 18S-VI | 0 | 0 | 0 | 0 |
| GD-ZJ19 | 2y | Zhanjiang | \ | ITS-A | 18S-VI | 12.5 | 0 | 0 | 0 |
| GD-ZJ20 | 2y | Zhanjiang | \ | ITS-A | 18S-VI | 12.5 | 0 | 0 | 0 |
| GD-ZJ21 | 2y | Zhanjiang | \ | ITS-A | 18S-VI | 0 | 0 | 0 | 0 |
| GD-ZJ22 | 2y | Zhanjiang | \ | ITS-A | 18S-VI | 0 | 0 | 0 | 0 |
| GD-ZJ23 | 2y | Zhanjiang | \ | ITS-A | 18S-VI | 12.5 | 0 | 0 | 0 |
| GD-ZJ24 | 2y | Zhanjiang | \ | ITS-A | 18S-VI | 50 | 50 | 50 | 50 |
| GD-ZJ25 | 2y | Zhanjiang | \ | ITS-A | 18S-VI | 0 | 0 | 0 | 0 |
| GD-ZJ26 | 2y | Zhanjiang | \ | ITS-A | 18S-VI | 12.5 | 0 | 0 | 0 |
| GD-ZJ27 | 2y | Zhanjiang | \ | ITS-A | 18S-VI | 12.5 | 0 | 0 | 0 |
| GD-ZJ28 | 2y | Zhanjiang | \ | ITS-A | 18S-VI | 0 | 0 | 0 | 0 |
| GD-ZJ29 | 2y | Zhanjiang | \ | ITS-A | 18S-VI | 0 | 0 | 0 | 0 |
| GD-ZJ30 | 2y | Zhanjiang | \ | ITS-A | 18S-VI | 0 | 0 | 0 | 0 |
| GD-ZJ31 | 2y | Zhanjiang | \ | ITS-A | 18S-VI | 0 | 0 | 0 | 0 |
| GD-ZJ32 | 2y | Zhanjiang | \ | ITS-A | 18S-VI | 0 | 0 | 0 | 0 |
| GD-ZJ35 | 2y | Zhanjiang | \ | ITS-A | 18S-VI | 12.5 | 12.5 | 0 | 0 |
| GD-ZJ40 | 2y | Zhanjiang | \ | ITS-A | 18S-VI | 0 | 0 | 0 | 0 |
| GD-ZJ41 | 2y | Zhanjiang | \ | ITS-A | 18S-VI | 0 | 0 | 0 | 0 |
| GD-ZJ42 | 2y | Zhanjiang | \ | ITS-A | 18S-VI | 0 | 12.5 | 25 | 0 |
| GD-ZJ43 | 2y | Zhanjiang | \ | ITS-A | 18S-VI | 12.5 | 0 | 0 | 0 |
